# Rift Valley Fever Virus Seroprevalence in Human Rural Populations of Gabon

**DOI:** 10.1371/journal.pntd.0000763

**Published:** 2010-07-27

**Authors:** Xavier Pourrut, Dieudonné Nkoghé, Marc Souris, Christophe Paupy, Janusz Paweska, Cindy Padilla, Ghislain Moussavou, Eric M. Leroy

**Affiliations:** 1 Institut de Recherche pour le Développement, UMR 190, Marseille, France; 2 Centre International de Recherches Médicales, BP 769, Franceville, Gabon; 3 Institut de Recherche pour le Développement, UMR 190, Mahidol University, Nakhon Pathom, Thaïland; 4 Institut de Recherche pour le Développement, UR 16, Montpellier, France; 5 Special Pathogens Unit, National Institute for Communicable Diseases, Johannesburg, South Africa; Tulane School of Public Health and Tropical Medicine, United States of America

## Abstract

**Background:**

Rift Valley fever (RVF) is a mosquito-borne viral zoonosis caused by a phlebovirus and transmitted by *Aedes* mosquitoes. Humans can also be infected through direct contact with blood (aerosols) or tissues (placenta, stillborn) of infected animals. Although severe clinical cases can be observed, infection with RVF virus (RVFV) in humans is, in most cases, asymptomatic or causes a febrile illness without serious symptoms. In small ruminants RVFV mainly causes abortion and neonatal death. The distribution of RVFV has been well documented in many African countries, particularly in the north (Egypt, Sudan), east (Kenya, Tanzania, Somalia), west (Senegal, Mauritania) and south (South Africa), but also in the Indian Ocean (Madagascar, Mayotte) and the Arabian Peninsula. In contrast, the prevalence of RVFV has rarely been investigated in central African countries.

**Methodology/Principal Findings:**

We therefore conducted a large serological survey of rural populations in Gabon, involving 4,323 individuals from 212 randomly selected villages (10.3% of all Gabonese villages). RVFV-specific IgG was found in a total of 145 individuals (3.3%) suggesting the wide circulation of Rift Valley fever virus in Gabon. The seroprevalence was significantly higher in the lakes region than in forest and savannas zones, with respective rates of 8.3%, 2.9% and 2.2%. In the lakes region, RVFV-specific IgG was significantly more prevalent in males than in females (respectively 12.8% and 3.8%) and the seroprevalence increased gradually with age in males but not in females.

**Conclusions/Significance:**

Although RVFV was suggested to circulate at a relatively high level in Gabon, no outbreaks or even isolated cases have been documented in the country. The higher prevalence in the lakes region is likely to be driven by specific ecologic conditions favorable to certain mosquito vector species. Males may be more at risk of infection than females because they spend more time farming and hunting outside the villages, where they may be more exposed to mosquito bites and infected animals. Further investigations are needed to determine the putative sylvan cycle of RVFV, including the mosquito species and the reservoir role of wild animals in the viral maintenance cycle.

## Author Summary

Rift Valley fever (RVF) is a disease transmitted by a mosquito bite (*Aedes*). Humans can also be infected through direct contact with blood (aerosols) or tissues (placenta, stillborn) of infected animals. Although severe clinical cases can be observed, infection with RVF virus (RVFV) in humans in most cases causes a febrile illness without serious symptoms. In small ruminants RVFV mainly causes abortion and neonatal death. RVFV distribution has been poorly investigated in Central Africa. We conducted a large scale serological survey of RVF antibodies in rural populations in Gabon, involving 4,323 individuals from 212 randomly selected villages. The results showed an overall RVFV prevalence of 3.3%, with values of 2.9% in the forested zones, 2.2% in savannas and 8.3% in the lakes region. These findings strongly suggest for the first time the wide circulation of Rift valley fever virus in Gabon and the possible existence of a sylvan cycle of RVF virus in this country. The serological higher prevalence in the lake region suggests that this region is likely to have particular ecological conditions, especially mosquito vector species, favoring the circulation of this virus. In Gabon, human cases of RVF may occur but are either misdiagnosed or not reported.

## Introduction

Rift Valley fever virus (RVFV) is a mosquito-borne RNA virus belonging to the *Phlebovirus* genus of the *Bunyaviridae* family. RVFV infects both humans and livestock [1]. Although severe clinical cases can be observed, infection with RVF virus (RVFV) in humans is, in most cases, asymptomatic or causes a febrile illness without serious symptoms. Some patients may develop serious complications, including meningoencephalitis (about 1%), hemorrhagic disorders (1%) and ocular disorders (retinitis and uveitis in 12% and about 30% respectively in Saudi Arabia) [2], [3], [4], [5]. The case fatality rate varied widely between different epidemics but ranged between 1% to 13%. RVFV induces abortion and stillbirth in small domestic ruminants, and has a major socio-economic impact in African countries [6], [7]. RVFV is transmitted by *Aedes* mosquitoes, but humans can also be infected through direct contact with blood (aerosols) or tissues (placenta, stillborn) of infected animals [8], [9].

RVFV was first isolated in Kenya in 1930 [10] and is now known to be widespread in many African countries, especially in non-forested regions. Until the 1970s, RVF was mainly reported in southern and eastern Africa (mainly Kenya), where it was considered as an animal disease, despite sporadic human cases [11]. After the 1970s, explosive outbreaks occurred in human populations throughout Africa, and principally in Egypt (1977–78, 1997–98) [2], [12], [13], Senegal and Mauritania (1987–1988) [14], [15], [16], Kenya, Somalia and Tanzania, (1997–1998, 2006–2007) [17], [18], Chad (2004) [19], Sudan (2008) [20] and South Africa (2010) [21], and also in the Arabian Peninsula (2000–2001) [22], Mayotte and Madagascar (2007–2008) [23], [24], [25]. In east Africa, RVF outbreaks coincided with heavy rainfall and local flooding, which can lead to expansion of vector populations [26], [27]. RVFV has been detected in many wild animal species (ungulates in Kenya, bats in Guinea, small vertebrates in Senegal and South Africa), but it is not known whether they serve to maintain the virus in the ecosystem during inter-epidemic periods, or whether they contribute to amplifying outbreaks [28], [29], [30], [31]. Although the RVFV cycle in savannas regions is now better understood, possible sylvan cycles in forested regions have not been explored [32].

In forested central Africa countries, no RVF outbreaks have been described, although RVFV-specific antibodies have been detected in wild animals and humans living in forested areas of Central African Republic (CAR) [28], [29], [30], [33], [34], [35], [36] and RVFV has been isolated from humans and wild mosquitoes (*Aedimorphus* and *Neolaniconion*) in the same regions [37], [38]. In Gabon, one of the most densely forested countries of central Africa, RVFV-specific antibodies were episodically detected in humans [35] but no systematic investigation has been conducted. We therefore undertook a large serological survey of RVFV in Gabon, focusing on human rural populations.

## Materials and Methods

### Studied population

**Figure 1:**
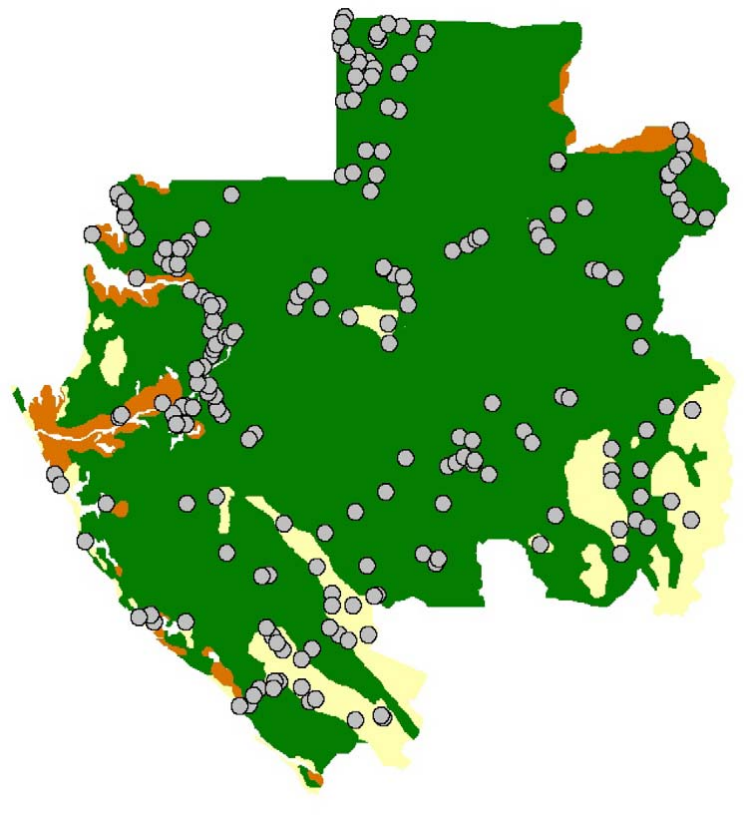
Location of the 212 villages (circles) sampled in the three main Gabonese ecosystems: forest (green), savannas (yellow) and lakes (brown).

Gabon is divided into nine provinces. Three-quarters of this country surface is covered by forest. Discontinuous areas of savannas are also found, mainly in the south and south-east of Gabon. In total, we collected 4323 serum samples from 212 villages (Figure 1), representing 10.7% of all villages in Gabon. No samples were collected in towns or cities. The required sampling population was calculated on the basis of on an estimated RVFV seroprevalence of 5%, and the villages were randomly selected (drawn lots, using a manual method) in the nine provinces. In each village, volunteers were interviewed and sampled. The survey was divided into nine time periods, corresponding to the survey of each province. We visited Estuaire province in July 2005, Moyen Ogooué in January 2006, Woleu-Ntem in April 2006, Ngounié in June 2006, Nyanga in January 2007, Haut-Ogooué in April 2007, Ogooué Ivindo in June 2007, Ogooué Lolo in September 2007 and Ogooué Maritime in May 2008. The villages were classified as located in forests, savannas, or lakes zones (or Lakeland) based on reference maps of Gabon. Forest regions were defined as dense and continuous forest, savannas as steppes or forest gallery, and the lakes zone as forested swamps, lagoons and lakes. In Gabon, the most humid region is the lakes zone with an annual rainfall about 2,000 mm and a tropical transitional humid climate.

### Data and blood collection

The following data were collected for each participant, using a standard questionnaire: demographics (age, sex, marital status), socio-environmental conditions (main occupation, contact with the forest, contact with animals, eating habits) and health (physical examination, last disease and symptoms). CIRMF (Centre International de Recherches Médicales de Franceville) has approved this study and the research. Informed consent was written and was obtained from all participants to this survey. A special authorization was delivered by Le Secrétaire Général du Ministère de la Santé Publique, lettre 00093/MSP/SG/SGAQM du 15/03/2006. Blood samples were collected directly in 7-ml EDTA Vacutainer tubes. Serum was separated each evening by centrifugation (2000 *g*), stored at −20°C in cryovials (VWR, Prolabo, France) and transported to CIRMF (Centre de Recherches Médicales de Franceville) for laboratory analysis.

### Serum analysis

The sera were tested with the RVFV sandwich enzyme-linked immunoassay (ELISA) developed and extensively validated by the National Institute for Communicable Diseases, Sandringham, South Africa [39]. The test uses RFVF antigen obtained from the whole virus to detect anti-RVFV IgG antibodies in human samples. Briefly, ELISA plates (Maxisorp, Nunc, Denmark) were coated with mouse anti-RVFV capture antibodies diluted 1∶10 000 in PBS overnight at +4°C. RVFV antigen diluted 1∶500 in 2% skimmed milk in PBS was then added to the wells. A mock antigen diluted in the same conditions was used as a control. The test and control sera were diluted 1∶400. Four high positive controls, two low positive controls and two negative controls were used for each plate. The specific activity of each serum (net optical density - OD) was measured by subtracting the OD of the sample and control wells. The mean net OD was calculated for the high positive control serum and the reactivity of each serum was calculated as percentage positivity (PP) of the high positive control serum, as follows:

PP serum = 100* net OD serum/mean net OD high positive control. Sera with PP values≥18 were considered positive and those with values of 17 or below were considered negative. This ELISA method has been validated against a serum neutralisation test [39].

### Statistical analysis

Statistical analysis was used to analyze the distribution of RVFV-specific IgG positive samples among the sampled population, and to determine risk factors and association between factors. We used comparisons of means or frequencies across the two groups (cases and non-cases) with Chi-Square test, T test, or non-parametric tests by MC simulation, and we analyze the association between RVFV-IgG and age with linear regression. We calculate odds ratios (OR) for exposure factors, and adjusted OR with possible confounding factors (age, gender, ecosystem), with adjusted Mantel-Haenszel Chi-square test for significance. Multivariate models stratified by ecosystem were constructed (logistic regression), which included univariate analysis of risk factors with significance level of ≤0.10, and the backwards stepwise elimination procedure was applied. Odds Ratios (OR) and exact 95% confidence intervals (CI) were used to access the association between risk factors and RVFV IgG seroprevalence. Statistical significance at 0.05 risk was used in tests and for confidence intervals (CI). Finally, we used 0.005 for risk factors analysis to take in account a Bonferroni correction for multitesting. We used STATA 10.0 software (College Station, Texas USA), Epi Info software (6.04, Epiconcept), SavGIS software (9.05, IRD).

## Results

### Characteristics of the population and overall RVFV specific IgG prevalence

**Figure 2:**
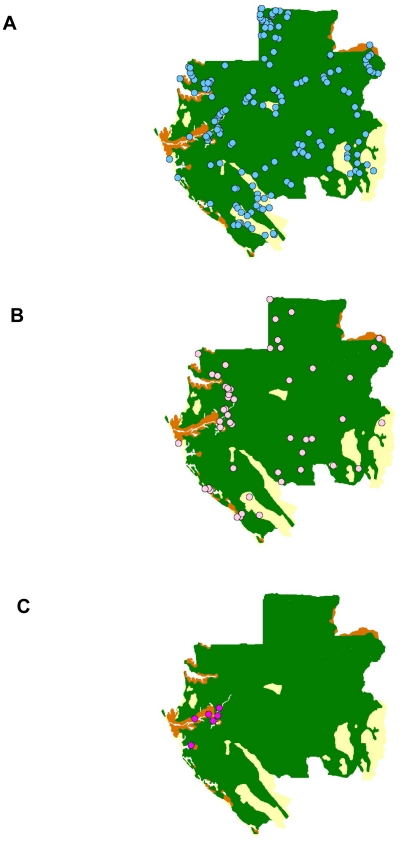
Location of the villages and levels of prevalence: low (A: 0–4%), intermediate (B: 5–14%) and high (C: >15%) RVFV antibody prevalence rates.

A total of 4323 villagers were interviewed with a mean age of 46.9 years (range 15–90 years) (Table 1). Females comprised 52.9% and males 47.1% of the total. Seventy six percent of the study participants lived in forested areas, 13% in savanna, and 11% in the lake region. The overall RVFV-specific IgG seroprevalence was 3.3%, with 4.3% in males and 2.5% in females. Seroprevalence was highest in the lake area (8.3%), followed by the savanna (2.9%), and the forest (2.2%). Hunters had the highest seroprevalence of those tested at 4.4%, followed by 3.5% in farmers and 2.4% for other professions combined. In the 212 surveyed villages (Figure 1), the RVFV IgG seroprevalence rates varied from 0 to 38%. Three levels of seroprevalence rates were defined: low (0–4%), intermediate (5–15%) and high (>15%) (Figure 2A, 2B, 2C). So, 155 (73%) villages were at low level, 51 (24%) at intermediate level and six (3%) at high level of RVFV IgG seroprevalence (Table 2). In forest and savanna areas, respectively 75% and 87% of the villages were at low level and respectively 24% and 13% at intermediate level. In the lakes region, the RVFV IgG prevalence was >5% in 14 villages (56%) and >15% in 6 villages (24%). Two villages among them (Pointe Elyse and Bordeaux) had high rates, respectively 27% (4/15) and 38% (5/13).

**Table 1:** Sociodemographic characteristics and RVFV IgG prevalence in the studied population of Gabon.

		Number of individuals	NumberRVFV IgG+	Prevalence (%)
**All**		4323	145	3.3
**Sex**	Male	2038	88	4.3
	Female	2285	57	2.5
**Age**	[15–33[	867	16	1.8
	[33–44[	851	30	3.5
	[44–54[	896	27	3
	[54–61[	959	35	3.6
	[61–90]	750	35	4.7
**Occupation**	Hunters	435	19	4.4
	Others	867	21	2.4
	Farmers	3021	105	3.5
**Ecosystem**	Forest	3312	95	2.9
	Savanna	551	12	2.2
	Lake	460	38	8.3

**Table 2:** RVFV IgG prevalence in the 212 villages of Gabon, classified by ecosystem.

	Region and number of villages (% per region)			
IgG prevalence (%)	Lakes	Forests	Savannas	Total
**0–4**	11 (44)	118 (75)	26 (87)	155 (73)
**5–14**	8 (32)	39 (24)	4 (13)	51 (24)
**>15**	6 (24)	0	0	6 (3)
**Total**	25 (100)	157 (100)	30 (100)	212 (100)

### Analysis of risk factors

Only the age, the gender and the ecosystem allowed rejecting the NULL hypothesis (of no significant relationship with RVFV-specific IgG prevalence). In particular, no statistical significance was found according to the activity of the villagers. We calculate ORs with confidence intervals, and p-value of the Chi-Square in a case-exposure test. Results are shown in Table 3.

**Table 3:** RVFV-IgG prevalence risk analysis for age, gender, ecosystem, occupation.

		Mean or frequency in positive samples	Mean or frequency in negative samples	p-value	OR	p-value (Chi-Square or T test)
**Gender**						
	**Male**	60.69%	46.74%	0.00032	1.75 [1.25,2.46]	0.00089
	**Female**	39.31%	53.28%	0.00035	0.57 [0.41,0.80]	0.00089
**Age**		50.68%	46.85%	0.0005		0.0006
	**[15–33[**	11.03%	20.31%	0.0024	0.49 [0.29, 0.83]	0.0058
	**[33–44[**	20.68%	19.69%	0.37	1.04 [0.69 , 1.57]	0.75
	**[44–54[**	18.62%	20.91%	0.24	0.87 [0.57 , 1.34]	0.52
	**[54–61[**	24.13%	22.20%	0.28	1.11 [0.76 , 1.64]	0.56
	**[61–90]**	25.52%	17.18%	0.0029	1.69 [1.16 , 2.48]	0.0082
**Ecosystem**						
	**Forest**	65.5%	77.0%	0.0004	0.57 [0.40, 0.80]	0.0013
	**Lakes**	26.2%	10.1%	<0.00001	3.20 [2.18 , 4.70]	<0.00001
	**Savanna**	8.3%	12.9%	0.05	0.60 [0.33 , 1.09]	0.10
**Occupation**						
	**Farmer**	72.41%	69.73%	0.23	1.12 [0.78 , 1.59]	0.50
	**Hunter**	13.10%	9.97%	0.10	1.36 [0.83 , 2.23]	0.21
	**Others**	14.48%	20.26%	0.04	0.67 [0.42 , 1.07]	0.09

Confident Intervals of Odd-Ratios correspond to 5% risk.

**Figure 3:**
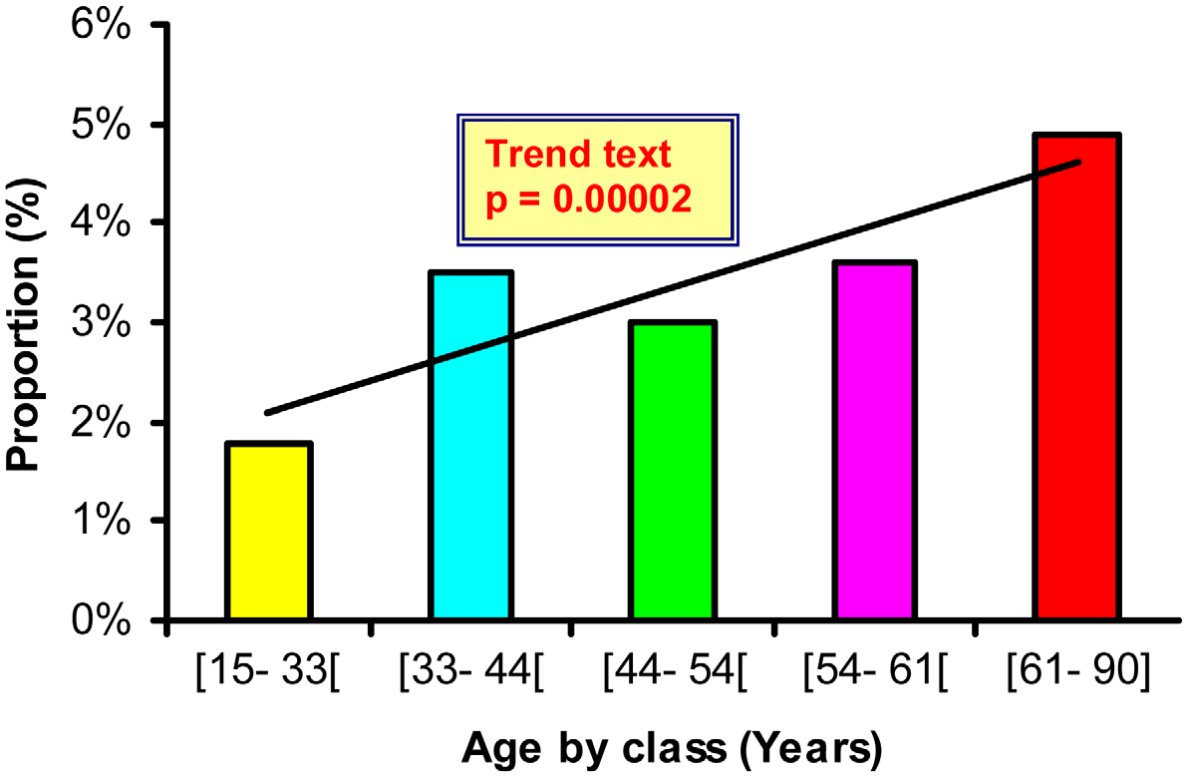
RVFV specific IgG seroprevalence according to the age class in Gabonese population.

Age factor: the age mean in the RVFV IgG positive group was 50.7 and 46.8 in the negative one (p<0.001). We class the age in 5 categories, near quintiles ([15–33[, [33–44[, [44–54[, [54–61[, [61–90]) for ORs and risk analysis. A significant (p = 0.00002) linear increase of RVFV IgG prevalence was noted between ordered age groups (Figure 3), even with a Bayesian adjustment (EBE) to reduce the variability difference between the groups.

Gender factor: gender shows a strong relationship RVFV specific IgG. The prevalence was higher in the male group (4.32%, OR = 1.75, [1.25, 2.46], Chi-Square = 11.05, p = 0.00089), than in the female group (2.50%, OR = 0.57, [0.41, 0.80], Chi-Square = 11.05, p = 0.00089). In order to highlight a possible confounding factor, adjusted OR with ecosystem or age were performed. These ORs did not show a significant difference (1.68 and 1.59 for the males and 0.59 and 0.62 for the females), showing that age distribution in positive samples has same distribution whatever the gender.

Ecosystem factor: ecosystem shows a strong OR difference (Table 3), with high risk in the lake ecosystem (OR = 3.2). Adjusted ORs with age and/or gender don't show any confounding factors (Table 4).

**Table 4:** Analysis of RVFV-IgG seroprevalence in the three ecosystems of Gabon with possible confounding factors: gender, age and a combination of age and gender.

		OR (Forest)	OR (Lakes)	OR (Savanna)
**All**		0.57 [0.40 , 0.80]	3.20 [2.18 , 4.70]	0.60 [0.33 , 1.09]
**Age**				
	**[15–33]**	0.45 [0.17,1.25]	3.60 [1.23,10.58]	0.60 [0.08 , 4.61]
	**[33–44]**	0.90 [0.39,2.03]	1.43 [0.49, 4.19]	0.89 [0.30 , 2.59]
	**[44–54]**	1.13 [0.45,2.84]	1.66 [0.56, 4.92]	0.45 [0.11 ,1.93]
	**[54–61]**	0.49 [0.24,0.99]	4.27 [1.86, 9.79]	0.87 [0.33 , 2.28]
	**[61–90]**	0.34 [0.17,0.66]	5.22 [2.64,10.30]	-
**Adjusted Age**		0.55 [0.39, 0.79]	3.37 [2.25 , 5.04]	0.75 [0.41 , 1.39]
**Gender**				
	**Female**	0.96 [0.52,1.76]	1.69 [0.82 , 3.49]	0.60 [0.24 , 1.52]
	**Male**	0.41 [0.27,0.64]	4.44 [2.78, 7.09]	0.62 [0.28 , 1.36]
**Adjusted Gender**		0.55 [0.38, 0.78]	3.34 [2.25 , 4.95]	0.61, [0.34 , 1.11]
**Adjusted Age and Gender**		0.53 [0.37, 0.76]	3.83 [2.52 , 5.81]	0.84, [0.45 , 1.55]

### Study of risk factors per ecosystem

In the forest and savanna areas, no significant relationship with RVFV-specific IgG prevalence and possible risk factors were found, except the activity (to be hunter, in these two ecosystems). The results are shown in Table 5. However, in the lake ecosystem, gender was a very high risk factor (OR = 4.44). Similarly than in the global population, the RVFV IgG prevalence rate increased with age. No other risk factor was highlighted.

**Table 5:** Odd ratios and adjusted odds ratios with possible confounding factors for the presence of RVFV antibodies according to potential risk factors, stratified by ecosystem, in Gabon.

Region		Variables	IgG (%)	OR	Adjusted OR (Age)	Adjusted OR (Gender)
**Forest**	**Gender**	Female	2.47%	0.74 [0.49 , 1.12]	0.77[0.51, 1.16]	-
		Male	3.31%	1.34 [0.89 , 2.02]	1.30 [0.86 , 1.96]	-
	**Age**	[15–33[	1.48%	0.45 [0.23 , 0.87]	-	0.46 [0.24 , 0.89]
		[33–44[	3.44%	1.24 [0.76 , 2.01]	-	1.25 [0.77 , 2.03]
		[44–54[	3.10%	1.11 [0.68 , 1.81]	-	1.13 [0.69 , 1.85]
		[54–61[	2.96%	1.05 [0.65 , 1.70]	-	1.05 [0.65 , 1.71]
		[61–90]	3.47%	1.30 [0.79 , 2.15]	-	1.28 [0.78 , 2.12]
	**Occupation**	Farmer	2.92%	1.06 [0.68 , 1.65]	0.95 [0.61 , 1.49]	1.23 [0.75 , 2.01]
		Hunter	3.64%	1.31 [0.71 , 2.43]	1.97 [1.03 , 3.76]	1.13 [0.59 , 2.18]
		Others	2.33%	0.78 [0.45 , 1.34]	1.02 [0.59 , 1.77]	0.72 [0.41 , 1.25]
**Lakes**	**Gender**	Female	3.83%	0.27 [0.13 , 0.59]	0.30 [0.13 , 0.71]	-
		Male	12.89%	3.66 [1.69 , 7.93]	3.29 [1.42 , 7.65]	-
	**Age**	[15–33[	4.85%	0.52 [0.20 , 1.36]	-	0.69 [0.25 , 1.92]
		[33–44[	4.76%	0.49 [0.17 , 1.41]	-	0.43[0.151, 1.26]
		[44–54[	4.54%	0.48 [0.17 , 1.39]	-	0.64 [0.21 , 1.93]
		[54–61[	11.43%	1.58 [0.69 , 3.60]	-	1.41 [0.60 , 3.30]
		[61–90]	14.78%	2.62 [1.33 , 5.16]	-	2.97 [1.48 , 5.94]
	**Occupation**	Farmer	9.86%	1.99 [0.89 , 4.44]	1.37 [0.58 , 3.22]	2.56 [1.14 , 5.77]
		Hunter	8.10%	1.01 [0.30 , 3.47]	1.99 [0.53 , 7.53]	0.58 [0.17 , 2.02]
		Others	4.20%	0.41 [0.16 , 1.08]	0.98 [0.33 , 2.90]	0.42 [0.16 , 1.11]
**Savanna**	**Gender**	Female	1.62%	0.56 [0.17 , 1.78]	0.73 [0.22 , 2.44]	-
		Male	2.89%	1.79 [0.56 , 5.72]	1.37 [0.41 , 4.61]	-
	**Age**	[15–33[	1.12%	0.50 [0.06 , 3.91]	-	1.32[0.15, 11.38]
		[33–44[	3.12%	1.71 [0.51 , 5.77]	-	1.75 [0.51 , 5.96]
		[44–54[	1.54%	0.64 [0.14 , 2.95]	-	0.66 [0.14 , 3.08]
		[54–61[	3.42%	1.90 [0.59 , 6.07]	-	1.78 [0.55 , 5.70]
		[61–90]	0%	0	-	-
	**Occupation**	Farmer	1.90%	0.60 [0.18 , 2.01]	0.45 [0.11, 1.88]	0.59 [0.13 , 2.70]
		Hunter	5.80%	3.75 [1.10, 12.82]	4.59 [1.06, 19.79]	3.64[0.80, 16.72]
		Others	0%	0	-	-

### Logistic regression analysis

Finally, multivariate analyses were performed by ecosystem. Only in the lakes region, gender and age remained significantly associated with a positive RVFV- IgG, with respectively adjusted OR = 3.85 [1.75–8.33], p = 0.001 and adjusted OR = 1.03 [1.01–1.059, p = 0.01 (Table 6).

**Table 6:** Adjusted seroprevalence and odds ratios in logistic regression for the presence of RVFV antibodies according to potential risk factors stratified by ecosystem, in Gabon.

Ecosystem	Risk factor	Adjusted OR	95%CI	P value
**Forest**	Gender	1.31	0.86–1.97	0.2
	Age	1.10	0.99–1.03	0.07
**Savanna**	Gender	1.82	0.56–5.88	0.32
	Age	1.00	0.96–1.05	0.97
**Lakes**	Gender	3.85	1.75–8.33	0.001
	Age	1.03	1.01–1.05	0.01

## Discussion

In this large serological survey, covering 10.7% of all Gabonese villages, we found an overall RVFV-specific IgG prevalence rate of 3.3%. This is surprisingly high for a country in which no RVFV outbreaks have ever been reported. As in Central Africa Republic, where no RVFV outbreaks have been described, successive serological surveys of people living in forested areas, in 1979–82, 1984–85 and 1994–97, showed RVFV-specific IgG prevalence rates ranging from 1.2% to 6.9% [33], [35], [36].

In countries with documented Rift Valley fever epidemics, the RVFV-specific IgG prevalence rates, measured in outbreak areas, were as high as 32% in Kenya in 1997 [17], 22.3% in Senegal in 1989 [16] and 24.4% in Mauritania in 1998 [40]. During interepidemic periods in Kenya, the RVFV IgG prevalence rates ranged from 1% to 19% [9], [41]. In Tanzania, a 2004 study showed a RVFV-specific IgG prevalence rate of 4% [42]. Thus, these data registered during interepidemic periods from epidemic countries are similar to those found in Gabon.

This result strongly suggests widespread circulation of Rift valley fever virus in Gabon even if the epidemiological cycle and the modalities of this circulation remain unknown. Classically, the RVFV cycle involves domestic animals (livestock) as viral amplifying hosts before transmission to humans, as has been shown in many African countries [7]. However, in Gabon, cattle herds are rare (except around major cities) and, in the rural areas where our investigations were carried out, few domestic animals such as cows, sheep and goats were found. Thus, in Gabon, the RVFV cycle may involve wild rather than domestic animals. This is supported by the isolation of RVFV from a specific forest mosquito, *Aedes (Neomelaniconion) gr. Palpalis* [38], in the Central African Republic and the detection of IgG in pygmies living in regions of this country where domestic animals are virtually absent [34].

One possible reason for the lack of reported RVF outbreaks or even isolated cases in Gabon is that cases of RVF might be attributed to malaria. Alternatively, less virulent strains may be circulating in Gabon. Although cross serological reactions with antibodies against unknown phleboviruses cannot be definitively ruled out, serological cross reactions against known phleboviruses are unlikely as the commercial ELISA method used in this survey has been extensively validated against a serum neutralisation test and was shown to be highly sensitive and specific for routine testing of human samples [39].

As RVFV transmission may vary with the local environment and vector distribution [41], we analyzed the results according to the principal ecosystems found in Gabon. We found a significantly higher RVFV IgG prevalence in the lakes region (8.2%) than in forest (2.9%) and savanna (2.1%) areas.

The lakes region is the most humid region of Gabon and is mainly composed of swamps and forested lagoons. This ecological situation, with omnipresent surface waters, could favor various mosquito species (or higher densities than elsewhere). A relationship has already been found between RVFV circulation and water resources in Kenya [41], Egypt [42], Senegal [43] and Saudi Arabia [44]. However, in Gabon, further investigation is needed to identify the mosquito species involved in RVFV transmission. In this ecosystem, the RVFV IgG prevalence was significantly higher (OR = 4.44, Table 4) in males (12.8%) than females (2.8%), and increased regularly with age (Table 6, Figure 3).

In the lakes region, males spend a large part of the day outside their villages, engaged in agriculture or hunting (or fishing). In contrast, females spend less time in agricultural activities and do not hunt; they remain indoors the rest of the time, cooking and taking care of infants. Thus, the higher RVFV IgG prevalence in males may be due to higher exposure to RVFV vectors or to infected animals. A similar gender difference was found in Kenya in 2006, where males had an RVFV IgG prevalence rate more than three times higher than females [41]. The reasons of the RVFV IgG seroprevalence increase according to age are unknown. This could be compatible with a continuous exposure of Gabonese populations to RVFV but also to a higher rate of exposition to mosquito vector bites infected with RVFV of the older age groups.

In conclusion, this first large serological survey of RVFV in central Africa strongly suggests that the virus circulates widely in Gabon, despite the lack of reported outbreaks. The overall high RVFV seroprevalence observed in Gabon suggests that human cases of RVF may occur but are either misdiagnosed or not reported. However, cross serological reactions with unknown phleboviruses cannot be ruled out. Further investigations are needed to isolate the Rift valley virus from human, animal or mosquito samples, to investigate the putative sylvan cycle of RVFV, and particularly to identify the mosquito species involved in human transmission and the potential role of wild animals as reservoir.

## Supporting Information

Checklist S1 STROBE Checklist
(0.10 MB DOC)

## References

[1] Bishop DH, Calisher CH, Casals J, Chumakov MP, Gaidamovich SY (1980). Bunyaviridae.. Intervirology.

[2] Laughlin LW, Meegan JM, Strausbaugh LJ, Morens DM, Watten RH (1979). Epidemic Rift Valley fever in Egypt: observations of the spectrum of human illness.. Trans R Soc Trop Med Hyg.

[3] McIntosh BM, Russell D, dos Santos I, Gear JH (1980). Rift Valley fever in humans in South Africa.. S Afr Med J.

[4] Madani TA, Al-Mazrou YY, Al-Jeffri MH, Mishkhas AA, Al-Rabeah AM (2003). Rift Valley fever epidemic in Saudi Arabia: epidemiological, clinical, and laboratory characteristics.. Clin Infect Dis.

[5] Al-Hazmi A, Al-Rajhi AA, Abboud EB, Ayoola EA, Al-Hazmi M (2005). Ocular complications of Rift Valley fever outbreak in Saudi Arabia.. Ophthalmology.

[6] Easterday BC, Mc GM, Rooney JR, Murphy LC (1962). The pathogenesis of Rift Valley fever in lambs.. Am J Vet Res.

[7] Coetzer JA (1982). The pathology of Rift Valley fever. II. Lesions occurring in field cases in adult cattle, calves and aborted foetuses.. Onderstepoort J Vet Res.

[8] Flick R, Bouloy M (2005). Rift Valley fever virus.. Curr Mol Med.

[9] LaBeaud AD, Ochiai Y, Peters CJ, Muchiri EM, King CH (2007). Spectrum of Rift Valley fever virus transmission in Kenya: insights from three distinct regions.. Am J Trop Med Hyg.

[10] Daubney R, Hudson JR, Garnham PC (1931). Enzootic hepatitis of Rift Valley fever; an undescribed virus disease of sheep, cattle and man from East africa.. Journal of Pathology and Bacteriology.

[11] Meegan JM, Bailey CL, Monath TP (1988). Rift Valley fever.. Arboviruses: epidemiology and ecology.

[12] Abd el-Rahim IH, Abd el-Hakim U, Hussein M (1999). An epizootic of Rift Valley fever in Egypt in 1997.. Rev Sci Tech.

[13] Sellers RF, Pedgley DE, Tucker MR (1982). Rift Valley fever, Egypt 1977: disease spread by windborne insect vectors?. Vet Rec.

[14] Ksiazek TG, Jouan A, Meegan JM, Le Guenno B, Wilson ML (1989). Rift Valley fever among domestic animals in the recent West African outbreak.. Res Virol.

[15] Jouan A, Le Guenno B, Digoutte JP, Philippe B, Riou O (1988). An RVF epidemic in southern Mauritania.. Ann Inst Pasteur Virol.

[16] Wilson ML, Chapman LE, Hall DB, Dykstra EA, Ba K (1994). Rift Valley fever in rural northern Senegal: human risk factors and potential vectors.. Am J Trop Med Hyg.

[17] Woods CW, Karpati AM, Grein T, McCarthy N, Gaturuku P (2002). An outbreak of Rift Valley fever in Northeastern Kenya, 1997–98.. Emerg Infect Dis.

[18] Bird BH, Githinji JW, Macharia JM, Kasiiti JL, Muriithi RM (2008). Multiple virus lineages sharing recent common ancestry were associated with a Large Rift Valley fever outbreak among livestock in Kenya during 2006–2007.. J Virol.

[19] Ringot D, Durand JP, Toulou H, Boutin JP, Davoust B (2004). Rift Valley fever in Chad.. Emerg Infect Dis.

[20] Adam I, Karsany MS (2008). Case report: Rift Valley Fever with vertical transmission in a pregnant Sudanese woman.. J Med Virol.

[21] WHO (2010). Rift Valley fever in South Africa.

[22] Ahmad K (2000). More deaths from Rift Valley fever in Saudi Arabia and Yemen.. Lancet.

[23] Morvan J, Saluzzo JF, Fontenille D, Rollin PE, Coulanges P (1991). Rift Valley fever on the east coast of Madagascar.. Res Virol.

[24] Sissoko D, Giry C, Gabrie P, Tarantola A, Pettinelli F (2009). Rift Valley fever, Mayotte, 2007–2008.. Emerg Infect Dis.

[25] Morvan J, Rollin PE, Laventure S, Rakotoarivony I, Roux J (1992). Rift Valley fever epizootic in the central highlands of Madagascar.. Res Virol.

[26] Davies FG, Linthicum KJ, James AD (1985). Rainfall and epizootic Rift Valley fever.. Bull World Health Organ.

[27] Anyamba A, Chretien JP, Small J, Tucker CJ, Formenty PB (2009). Prediction of a Rift Valley fever outbreak.. Proc Natl Acad Sci U S A.

[28] Gora D, Yaya T, Jocelyn T, Didier F, Maoulouth D (2000). The potential role of rodents in the enzootic cycle of Rift Valley fever virus in Senegal.. Microbes Infect.

[29] Pretorius A, Oelofsen MJ, Smith MS, van der Ryst E (1997). Rift Valley fever virus: a seroepidemiologic study of small terrestrial vertebrates in South Africa.. Am J Trop Med Hyg.

[30] Oelofsen MJ, Van der Ryst E (1999). Could bats act as reservoir hosts for Rift Valley fever virus?. Onderstepoort J Vet Res.

[31] Evans A, Gakuya F, Paweska JT, Rostal M, Akoolo L (2008). Prevalence of antibodies against Rift Valley fever virus in Kenyan wildlife.. Epidemiol Infect.

[32] Chevalier V, de la Rocque S, Baldet T, Vial L, Roger F (2004). Epidemiological processes involved in the emergence of vector-borne diseases: West Nile fever, Rift Valley fever, Japanese encephalitis and Crimean-Congo haemorrhagic fever.. Rev Sci Tech.

[33] Meunier DM, Johnson ED, Gonzalez JP, Georges-Courbot MC, Madelon MC (1987). [Current serologic data on viral hemorrhagic fevers in the Central African Republic].. Bull Soc Pathol Exot Filiales.

[34] Nakounne E, Selekon B, Morvan J (2000). [Microbiological surveillance: viral hemorrhagic fever in Central African Republic: current serological data in man].. Bull Soc Pathol Exot.

[35] Gonzalez JP, Josse R, Johnson ED, Merlin M, Georges AJ (1989). Antibody prevalence against haemorrhagic fever viruses in randomized representative Central African populations.. Res Virol.

[36] Gonzalez JP, Mc Cormick JB, Saluzzo JF, Georges AJ (1983). Les fièvres hémorragiques africaines d'origine virale, contribution à leur étude en République Centrafricaine.. Cahiers ORSTOM.

[37] Digoutte JP, Jacobi JC, Robin Y, Gagnard VJ (1974). [Zinga virus infection in man].. Bull Soc Pathol Exot Filiales.

[38] Cordellier R, Geoffroy B (1976). Les moustiques de République Centrafricaine: Distribution, abondance et fréquence des Culicinés dans l'ouest du pays. Les arbovirus isolés.. Travaux et documents de l'ORSTOM.

[39] Paweska JT, Burt FJ, Swanepoel R (2005). Validation of IgG-sandwich and IgM-capture ELISA for the detection of antibody to Rift Valley fever virus in humans.. J Virol Methods.

[40] Nabeth P, Kane Y, Abdalahi MO, Diallo M, Ndiaye K (2001). Rift Valley fever outbreak, Mauritania, 1998: seroepidemiologic, virologic, entomologic, and zoologic investigations.. Emerg Infect Dis.

[41] LaBeaud AD, Muchiri EM, Ndzovu M, Mwanje MT, Muiruri S (2008). Interepidemic Rift Valley fever virus seropositivity, northeastern Kenya.. Emerg Infect Dis.

[42] Swai ES, Schoonman L (2009). Prevalence of Rift Valley Fever Immunoglobulin G Antibody in Various Occupational Groups Before the 2007 Outbreak in Tanzania.. Vector Borne Zoonotic Dis.

[43] Zeller HG, Fontenille D, Traore-Lamizana M, Thiongane Y, Digoutte JP (1997). Enzootic activity of Rift Valley fever virus in Senegal.. Am J Trop Med Hyg.

[44] Elfadil AA, Hasab-Allah KA, Dafa-Allah OM (2006). Factors associated with rift valley fever in south-west Saudi Arabia.. Rev Sci Tech.

