# Supplementary material for: Rift Valley Fever Virus Seroprevalence in Human Rural Populations of Gabon
Source: PLoS Negl Trop Dis. 2010 Jul 27;4(7):e763. doi: 10.1371/journal.pntd.0000763 (PMC2910672; doi:10.1371/journal.pntd.0000763)
Supplement: Checklist S1 — STROBE Checklist (0.10 MB DOC) [file pntd.0000763.s001.doc]

STROBE Statement—Checklist of items that should be included in reports of ***cross-sectional studies***

|  | Item No | Recommendation |
| --- | --- | --- |
| **Title and abstract** | 1 | 1. Indicate the study’s design with a commonly used term in the title or the abstract   **Rift Valley fever (RVF) seroprevalence in Gabon** |
|  |  | 1. Provide in the abstract an informative and balanced summary of what was done and what was found   **We conducted a large serological survey of rural populations in Gabon, involving 4323 individuals from 212 randomly selected villages (10.3% of all Gabonese villages). RVFV-specific IgG was found in a total of 145 individuals (3.3%) suggesting for the first time the wide circulation of Rift valley fever virus in Gabon.** |
| Introduction | | |
| Background/rationale | 2 | Explain the scientific background and rationale for the investigation being reported  **RVF an important hemorrhagic disease has been investigated in many countries of Africa but poorly in the Central Africa region** |
| Objectives | 3 | State specific objectives, including any prespecified hypotheses  **Investigation of RVFV in Gabon** |
| Methods | | |
| Study design | 4 | Present key elements of study design early in the paper  **Seroprevalence (cross sectional) survey** |
| Setting | 5 | Describe the setting, locations, and relevant dates, including periods of recruitment, exposure, follow-up, and data collection  **Rural populations in 10.7% of the Gabonese villages, in all regions and all provinces; investigation from july 2005 to may 2008. Questionnaire including demographics (age, sex, marital status), socio-environmental conditions (main occupation, contact with the forest, contact with animals, eating habits) and health (physical examination, last disease and symptoms). Written informed consent obtained from all participants** |
| Participants | 6 | 1. Give the eligibility criteria, and the sources and methods of selection of participants   **Randomization of the villages; selection of the participants (volunteers) in the village** |
| Variables | 7 | Clearly define all outcomes, exposures, predictors, potential confounders, and effect modifiers. Give diagnostic criteria, if applicable  **Comparison of RVFV prevalence with age, sex and ecosystem (savannas, forest, Lakeland). The villages were classified as located in forests, savannas, or lakes zones based on reference maps of Gabon.** |
| Data sources/ measurement | 8* | For each variable of interest, give sources of data and details of methods of assessment (measurement). Describe comparability of assessment methods if there is more than one group |
| Bias | 9 | Describe any efforts to address potential sources of bias  **Randomization; logistic regression** |
| Study size | 10 | Explain how the study size was arrived at  **The required sampling population was calculated on the basis of on an estimated RVFV seroprevalence of 5% (using n = ε ² [p (1-p)] / e ²; with ε = 1.96 for a Confidence Interval CI at 95 %, e precision = 2 % and p = expected prevalence)** |
| Quantitative variables | 11 | Explain how quantitative variables were handled in the analyses. If applicable, describe which groupings were chosen and why  **Please see below** |
| Statistical methods | 12 | **Statistical comparisons of seroprevalence rates across different groups with the Chi1square test or Fisher test when appropriate. A trend test across ordered groups, were implemented for seroprevalence of RVFV by class of age. In the lakes region, variables were selected for inclusion in a multivariate model. Adjusted Odds ratios and 95% Cis were used to assess the association between sex, age and RVFV serostatus using STATA 10.0 software (College Station, Texas USA). Epi Info software (6.04, Epiconcept) were used and statistical significance was assumed at p < 0.05.** |
|  |  |  |
|  |  |  |
|  |  |  |
|  |  |  |
| Results | | |
| Participants | 13* | 1. Report numbers of individuals at each stage of study—eg numbers potentially eligible, examined for eligibility, confirmed eligible, included in the study, completing follow-up, and analysed   **In total, 4323 villagers were interviewed and sampled comprising 3312 (76%)143 persons in forested regions, 551 (13%) in savannas and 460 (11%) in the lakes region.** |
|  |  |  |
|  |  |  |
| Descriptive data | 14* | **In the lakes region, of the 460 persons interviewed, 225 were males and 235 females. The primary occupation (Table 3) was agriculture for 304 villagers (66% overall, 56% of males and 76% of females), hunting for 37 individuals (16% of males; no females) and others activities (sellers, officers, teachers or student) for 119 persons (28% of males and 24% of females).** |
|  |  |  |
| Outcome data | 15* | Report numbers of outcome events or summary measures |
| Main results | 16 | **The seroprevalence was significantly higher in the lakes region than in forest and savannas zones, with respective rates of 8.2%, 2.9% and 2.1%. In the lakes region, RVFV-specific IgG was significantly more prevalent in males than in females (respectively 12.8% and 3.8%) and the seroprevalence increased gradually with age in males but not in females.** |
|  |  |  |
|  |  |  |
| Other analyses | 17 | Report other analyses done—eg analyses of subgroups and interactions, and sensitivity analyses |
| Discussion | | |
| Key results | 18 | Summarise key results with reference to study objectives  **In this large serological survey, covering 10.7% of all Gabonese villages, we found an overall RVFV-specific IgG prevalence rate of 3.3%. This is surprisingly high for a country in which no RVFV outbreaks have ever been reported.** |
| Limitations | 19 | Discuss limitations of the study, taking into account sources of potential bias or imprecision. Discuss both direction and magnitude of any potential bias  **Although cross serological reactions with antibodies against unknown phleboviruses cannot be definitively ruled out, serological cross reactions against known phleboviruses are unlikely** |
| Interpretation | 20 | Give a cautious overall interpretation of results considering objectives, limitations, multiplicity of analyses, results from similar studies, and other relevant evidence  **This result strongly suggests widespread circulation of Rift valley fever virus in Gabon even if the epidemiological cycle and the modalities of this circulation remain unknown.** |
| Generalisability | 21 | Discuss the generalisability (external validity) of the study results |
| Other information | | |
| Funding | 22 | Give the source of funding and the role of the funders for the present study and, if applicable, for the original study on which the present article is based  **CIRMF is supported by the Government of Gabon, Total-**  **Fina-Elf Gabon, and the Ministère de la Coopération Française. This work was alsosupported by a Fonds de Solidarité Prioritaire grant from the Ministère des Affaires Etrangères de la France (FSP n° 2002005700)** |
